# Biomarkers for the Diagnosis of Alzheimer’s Disease in Clinical Practice: The Role of CSF Biomarkers during the Evolution of Diagnostic Criteria

**DOI:** 10.3390/ijms23158598

**Published:** 2022-08-02

**Authors:** Maciej Dulewicz, Agnieszka Kulczyńska-Przybik, Piotr Mroczko, Johannes Kornhuber, Piotr Lewczuk, Barbara Mroczko

**Affiliations:** 1 Department of Neurodegeneration Diagnostics, Medical University of Bialystok, 15-269 Bialystok, Poland; agnieszka.kulczynska-przybik@umb.edu.pl (A.K.-P.); piotr.lewczuk@uk-erlangen.de (P.L.); mroczko@umb.edu.pl (B.M.); 2 Department of Criminal Law and Criminology, Faculty of Law, University of Bialystok, 15-213 Bialystok, Poland; p.mroczko@uwb.edu.pl; 3 Department of Psychiatry and Psychotherapy, Universitätsklinikum Erlangen and Friedrich-Alexander Universität Erlangen-Nürnberg, 91054 Erlangen, Germany; johannes.kornhuber@uk-erlangen.de; 4 Department of Biochemical Diagnostics, Medical University of Bialystok, 15-269 Bialystok, Poland

**Keywords:** Alzheimer’s disease, biomarkers, clinical and research criteria

## Abstract

Alzheimer’s disease (AD) is a progressive condition and the most common cause of dementia worldwide. The neuropathological changes characteristic of the disorder can be successfully detected before the development of full-blown AD. Early diagnosis of the disease constitutes a formidable challenge for clinicians. CSF biomarkers are the in vivo evidence of neuropathological changes developing in the brain of dementia patients. Therefore, measurement of their concentrations allows for improved accuracy of clinical diagnosis. Moreover, AD biomarkers may provide an indication of disease stage. Importantly, the CSF biomarkers of AD play a pivotal role in the new diagnostic criteria for the disease, and in the recent biological definition of AD by the National Institute on Aging, NIH and Alzheimer’s Association. Due to the necessity of collecting CSF by lumbar puncture, the procedure seems to be an important issue not only from a medical, but also a legal, viewpoint. Furthermore, recent technological advances may contribute to the automation of AD biomarkers measurement and may result in the establishment of unified cut-off values and reference limits. Moreover, a group of international experts in the field of AD biomarkers have developed a consensus and guidelines on the interpretation of CSF biomarkers in the context of AD diagnosis. Thus, technological advancement and expert recommendations may contribute to a more widespread use of these diagnostic tests in clinical practice to support a diagnosis of mild cognitive impairment (MCI) or dementia due to AD. This review article presents up-to-date data regarding the usefulness of CSF biomarkers in routine clinical practice and in biomarkers research.

## 1. Introduction

Alzheimer’s disease (AD) is a chronic, progressive neurodegenerative disease that is the most common cause of dementia worldwide, accounting for an estimated 60% to 80% of all dementia cases [1]. However, it is essential to remember that AD is not a normal part of the ageing process and the ageing process in itself does not cause AD [1,2]. The neuropathological processes leading to AD begin many years before the onset of cognitive impairment, such as memory loss and language problems [3,4]. The first neuropathological hallmarks of the disorder are the accumulation and formation of amyloid β (Aβ) plaques, and intracellular neurofibrillary tangles (NFTs) composed of Tau protein [4,5,6,7]. Disrupted brain clearance and excessive production of plaque deposits can occur ~20 years before the onset of cognitive impairment [1,6,8,9]. Hyperphosphorylated-tau protein and NFTs can be detected 10–15 years prior to the onset of clinical symptoms [1,6,8,9]. Currently, fluid and imaging biomarkers are the most objective measures of neuropathological processes, allowing for a more accurate diagnosis and assessment of the risk of disease progression [6,10]. According to the most recently proposed diagnostic criteria for AD, diagnosis of the disease should rely on using in vivo biomarkers of amyloid pathology (decreased Aβ 1-42 or Aβ 1-42/Aβ 1-40 ratio in CSF, or increased tracer retention in amyloid positron emission tomography (PET)) and tau pathology (increased tracer retention in tau PET and increased CSF levels of tTau and pTau181), which allows for an earlier and more accurate diagnosis of the disease [4,5,11,12,13]. These two main groups of molecules are well established CSF biomarkers of AD pathology. Other AD biomarkers may also be used for early diagnosis; however, their role in amyloid pathology and AD genetics should be studied more thoroughly [14]. In clinical practice, cerebral glucose uptake (GU) measured by fluorodeoxyglucose positron emission tomography (FDG-PET) is also widely used. Neuroimaging tests detect not only brain metabolism, but also neuronal integrity.

An accurate diagnosis of AD commonly involves an interdisciplinary approach to evaluating the clinical signs and symptoms of this multifactorial disease and the biochemical changes [1,4,15]. Diagnostic criteria, recommendations, scoring systems and scales for in vivo biomarkers improve early diagnosis and monitoring of disease progression [6,16,17,18,19,20]. Scientists continue to search for the main and earliest triggers underlying the neurodegenerative changes associated with AD dementia [16]. Heterogeneous mechanisms may lead to the development of AD, which may also be reflected in cognitive, clinical and biochemical changes [1]. Considering the neurocognitive symptoms of AD, the most common clinical signs are memory loss and sometimes depression and apathy [6]. Middle-stage and later symptoms include disorientation, confusion, behavioral changes and problems with speech or language [6,16,17]. These symptoms also have a neurobiological basis and can be monitored based on the assessment of biological substances reflecting pathological changes in human fluids decades before disease onset [4,20]. It is postulated that, in addition to obtaining the patient’s medical history, several tests should be performed to assess decline of cognitive function related to AD, including neuropsychological tests, neuroimaging tests and assessment of biochemical markers [1,6]. CSF biomarkers are widely discussed in working groups and included in international guidelines for clinical practice [4,6,15,18,21,22]. Clinicians may encounter a number of challenges in diagnosing AD [20] due to mixed pathologies related to cerebrovascular disease or Lewy body dementia (LBD). Furthermore, the diagnostic process may be complicated because of the use of different diagnostic techniques or presence of other, pre-analytical factors [8,11,19,20,23,24,25]. Therefore, proper recognition of pre-analytical conditions will result in improved reproducibility and quality of CSF measurements. The pre-analytical factors that are of particular importance include the types of sample collection and storage tubes, storage temperature, delayed freezing of samples, long-term stability and the number of freeze–thaw cycles, contamination of CSF with blood, and the volume of storage samples. Moreover, since biomarker results were interpreted differently in different centers, which led to misunderstandings, attempts have been made to standardize the interpretation of CSF biomarker results with respect to the clinical picture of AD and MCI [4,6,15,16,18,21,22,26]. Despite the application of a number of established biomarkers in clinical practice, the search for new candidate biomarkers continues [27,28].

The main aim of the present paper was to discuss key issues relating to the biochemical diagnosis of AD in clinical practice. The review focuses primarily on AD spectrum, related CSF biomarkers and diagnostic criteria. The paper is not only a review of the available literature and diagnostic criteria, but also reports our own experience, research and international cooperation with diagnostic centers. Biomarkers from blood and other body fluids are not discussed in detail.

## 2. Molecular Neuropathology of Alzheimer’s Disease and Related Biomarkers

There are many theories attempting to explain AD dementia including the Aβ cascade, Tau pathology, neuroinflammation, cholinergic and oxidative stress hypotheses. The most extensively studied mechanisms of AD pathology are those related to the main pathological features of the disease—the formation of Aβ plaques and tau neurofibrillary tangles, found in the critical brain regions responsible for many cognitive functions.

Senile plaques are composed predominantly of aggregated β-amyloid [29]. The hydrophobic peptide of Aβ is released by enzymatic cleavage of APP by β-secretase and γ-secretase, which leads to the formation of Aβ peptides of several different lengths, including Aβ 1-42 [4]. However, of significance is Aβ peptide, ending with a C terminus at residue 42 (Aβ 1-42) [30,31]. Studies on brain tissue from AD patients have demonstrated that Aβ 1-42 is the main component of senile plaques [32]. There are many other isoforms of Aβ and, although Aβ1-40 is the most abundant (~90%), it is not a useful biomarker for differentiating between AD patients and cognitively normal controls. Several studies and meta-analyses have reported a reduced CSF concentration of Aβ 1-42 in AD patients, even in the preclinical phase of the disease [11,33,34]. However, it is still not well understood why Aβ 1-42 is decreased in the CSF of AD patients [35]; although, several hypotheses concerning this neuropathological conundrum have been proposed [4,36,37]. Furthermore, some authors suggest that CSF concentrations are reduced as a result of Aβ 1-42 sequestration in plaques. Other possible explanations are related to enhanced neuronal degradation, which leads to a reduction in the production Aβ 1-42; thereby causing its decreased concentrations in the CSF. However, this seems less probable since other isoforms should also be significantly downregulated. Fibrillogenesis is strictly related to the aggregation of Aβ 1-42 and Aβ 1-40. A recent study has demonstrated the effect of the combinations of monomers Aβ37, Aβ38 and Aβ 1-40 on the growth of Aβ fibrils [38]. The study revealed that smaller isoforms of Aβ (37 or 38) can aggregate by themselves and with longer forms. Aβ37 and Aβ38 take a longer time to transform into fibrils than Aβ 1-42 and Aβ 1-40, which transform by an autocatalytic secondary nucleation reaction [38]. Aβ 1-42 isoforms aggregate more rapidly than other isoforms, taking less than an hour, while shorter forms take several days to transform [38]. Smaller and more slowly fibrillating forms of Aβ have an inhibitory effect on the rate of senile plaque formation [38]. Furthermore, Aβ38 has an inhibitory effect on fibril formation, but the most significant effect was observed by the proportion of 1:3:2 or 1:4:1 of Aβ 1-40/Aβ38/Aβ37 [38]. This and other studies appear to indicate a therapeutic target related to γ-secretase modulators, which could reduce Aβ plaque formation [35,39,40]. There have been several promising attempts to use other conformations. It is important to note that the Aβ 1-42/Aβ 1-40 ratio improves the sensitivity and specificity of diagnosis compared to Aβ1–42 in CSF alone [11,41,42]. This is due to the distribution of a quotient (Aβ 1-42/Aβ 1-40) having smaller dispersion of the random variable in the numerator (Aβ 1–42) [43]. Above all, it seems reasonable that the most common form is compared to the one most involved in the pathology at all isoforms [4,43].

The tau proteins are a family of six well-established (but probably more) isoforms, which result from alternative splicing on the MAPT gene (microtubule-associated protein tau) located on chromosome 17 [44]. The physiological role of tau is stabilization and nucleation of neuronal microtubules; although it performs many other functions, such as broad cell signaling [37,44]. CSF total tau concentration has been extensively studied and interpreted as an unspecific biomarker of neuronal damage in neurodegenerative diseases [45,46]. Elevated tTau levels are observed in many diseases, such as AD, PD and a number of other tauopathies. Phosphorylation of tau protein can occur at 85 potential sites involving serine, threonine and tyrosine [47]. The phosphorylated forms of tau (pTau181, pTau217, pTau231, pTau235) appear to be more specific to AD and detectable in CSF and in plasma [45,48]. Different phosphorylation sites of tau modulate intracellular interactions and influence the intensity of various tau-dependent diseases (tauopathies) [47,49]. Moreover, tau exhibits increased phosphorylation (hyperphosphorylation) at selected sites (e.g., threonine pTau181) and aggregates into neuropathological forms of NFTs [50]. Elevated CSF levels of tau and pTau181 in MCI and cognitively normal adults are associated with a higher risk of developing AD dementia [51].

## 3. Characteristics of Diagnostic Criteria of AD Spectrum

**Figure 1:**
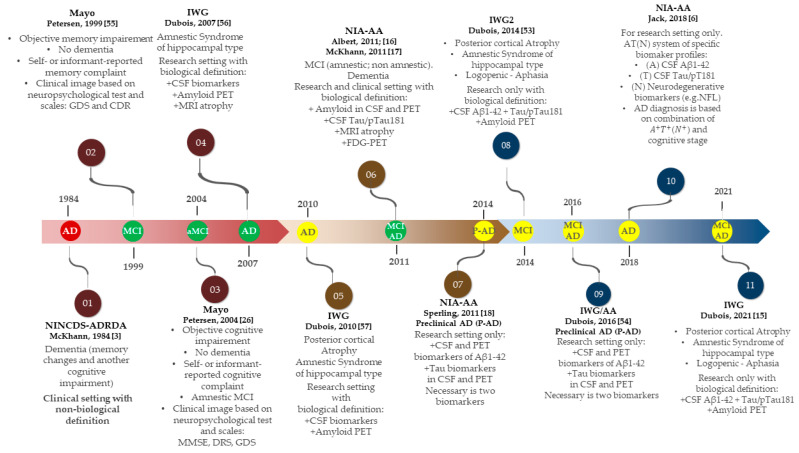
Evolution of diagnostic frameworks for Alzheimer’s disease [3,6,15,16,17,18,26,53,54,55,56,57]. Colors: Red—Diagnostic criteria that are no longer commonly used; Green—Widely accepted and used clinically and/or in research; Yellow—Used primarily in research and recommended for research use only. Abbreviations: GDS—Global Deterioration Scale, CDR—Clinical Dementia Rating Scale, DRS—Dementia Rating Scales; MMSE—Mini-mental state examination; PET—Positron emission tomography; FDG—Fluorodeoxyglucose.

The definition and diagnostic criteria of AD, as well as hypotheses on the pathogenesis of the disease, have changed over the years [22,27,52]. An evolution of the diagnostic criteria for AD has been driven by cooperation between clinicians and scientists (Figure 1) [3,6,15,16,17,18,26,53,54,55,56,57]. Since the first diagnostic criteria were published in 1984, many things have changed. The development of new research methods and a deeper understanding of the biological mechanisms of the disease have resulted in improvement in diagnostic criteria and progress in clinical trials [3,4,22,58]. Initially, AD was diagnosed only on the basis of clinical symptoms, which resulted in recognizing the disease at a late stage and did not allow for an accurate diagnosis. A milestone in diagnosing AD and MCI was the McKhann and Albert criteria published in 2011, in which biomarkers were considered one of the appropriate diagnostic methods [16,17]. These categories are among the most commonly used criteria in diagnosing MCI due to AD [16,17].

The application of CSF biomarkers in routine clinical practice allows for detection of the disease at a very early, asymptomatic (preclinical) stage through the prodromal phase (MCI—mild cognitive impairment) to full-blown, symptomatic AD [3,6,15,53]. Other consensus and research groups (e.g., IWG) have proposed diagnosing AD as a clinical and biological entity based on in vivo biomarkers [6,16,17,18,20,21]. Some of these criteria are still in research and development for later clinical use (yellow dots in Figure 1) [6,15,18,53,54,57]. By way of illustration, criteria for the preclinical stage are still in the development phase and are recommended only for research use (Figure 1) [6,15,18,54].

For a number of years, AD was defined only on the basis of symptoms, while currently CSF and MRI/PET biomarkers are applied in several diagnostic criteria (Figure 1). Biomarkers reflect different types of pathophysiology found in the brains of individuals with AD spectrum [4,27,53]. Firstly, AD biomarkers can aid in the clinical diagnosis of the disease, particularly when symptoms are inconclusive or uncharacteristic [1,15]. Secondly, biomarkers are essential components of clinical research that allow for studying the course of different pathologies over time [4,6,15,19,59]. There are several established biomarkers which have been standardized and validated for research on the AD spectrum [6,16,20,59]. Biomarkers enable us to observe temporal trends in pathology, prevalence and morbidity. Furthermore, biomarkers are also used in establishing differential diagnosis.

## 4. Diagnostic Scales for Interpretation of CSF Biomarker Profiles

CSF biomarkers include tTau, pTau181 and 42-amino acid β-amyloid isoform (Aβ 1-42) [11]. Many studies have consistently demonstrated that the majority of patients with a clinical diagnosis of AD exhibit a typical ‘AD biomarker profile’ consisting of elevated tTau and pTau181 values and decreased Aβ 1-42 levels [4,11]. Profiling or scoring of AD biomarkers is both useful and effective as it facilitates biomarker interpretation and allows for the comparison of results with other research or test centers [6,20,60,61,62]. The significance of CSF biomarkers in diagnosing AD and other types of dementia is well established. However, problems with interpretation may sometimes arise, particularly when not all biomarkers are pathological. Then, a question of how to use these data, which are often heterogeneous, arises. One of the proposed solutions is using the probability scale to assess if pathological processes characteristic of AD are occurring in the patient with cognitive impairment. A practical example of the application of such a scale is the Erlangen Score algorithm [61]. The final score, which may confirm AD pathology, is obtained by adding the results from CSF biomarkers, including Aβ 1-42 biomarkers (0 = normal; 1 = borderline pathological; 2 = pathological) and Tau/pTau biomarkers (0 = normal; 1 = borderline pathological; 2 = pathological) based on the cut-off values accepted in the laboratory [63,64]. The result is a total score that can be interpreted as: 0—neurochemically normal; 1—AD neurochemically improbable; 2–3—AD neurochemically possible; 4—AD neurochemically probable [60,61]. Furthermore, the algorithm is optimized for very high Tau values, which indicate a rapid progression of neurodegenerative changes (e.g., Creutzfeldt–Jakob Disease (CJD)) [4]. By way of illustration, AD is scored at 4 due to the pathological status of both biomarkers (Aβ 1-42 (2) + Tau/pTau (2) = 4). In general, patients with scores of 2 and 3 can be classified as MCI due to AD. However, caution should be exercised when interpreting results typical for MCI due to AD since several interactions in the scoring system are possible. A significant impact on the final score is made by the border zone [19,61]. The border zone is generally defined as a pathological result within 10% of the reference value, i.e., a 10% decrease in Aβ 1-42 and/or Aβ 1-42/Aβ 1-40, or a 10% increase in Tau and/or pTau181 [61]. Using this 10% margin for biomarker results makes this algorithm more sensitive to changes in measurement of concentrations [61]. A number of centers around the world, and particularly in Europe, use the Erlangen Scale not only in research, but also in routine diagnostics [19].

The ATN (amyloid, Tau, neurodegeneration) classification system allows for categorization of individuals based on biomarkers indicative of neurodegenerative pathology [6]. The name of system is an acronym formed from the initial letters of the following words: amyloid (CSF Aβ or amyloid PET: “A”), hyperphosphorylated tau (CSF p-tau or tau PET: “T”) and neurodegeneration (atrophy on structural MRI, FDG PET or total Tau in CSF: “N”), resulting in nine different combinations of biomarkers [6]. Each biomarker category is rated as positive or negative. Moreover, the International Working Group (IWG) has developed and recommended this rating system [6]. The results of the positive and negative biomarker profiles are categorized into three groups: “Normal AD biomarkers”, “Alzheimer’s continuum” with four subcategories, and “Non-AD pathological change”. According to this scale, AD pathology may be recognized based on the following pattern of biomarkers: A+T+(N−) or A+T+(N+), and criteria for the control group are based on: A-T-(N)− [6,62]. The ATN system and Erlangen Score are open to new biomarker categories, which is highly desirable in regard to new candidates for biomarkers. There are several potentially significant categories of biomarkers reflecting different pathological aspects, which could be related to: synaptic, metabolic, pericyte or axonal injury [63,64,65,66]. The innovative idea to add “X” category to the ATN framework was presented by Hampel et al. [67]. The addition of the X category to the ATN framework allows for a better understanding of other pathologies and dynamic changes with the development of AD [67]. Huang et al. proposed division by the X category for two subcategories, which could better reflect a whole spectrum of pathology in the central nervous system (CSN) $X_{c}$ and in periphery $X_{p}$ [68]. In $X_{c}$, authors focused on biomarkers related to synaptic damage, glial cells, neuroinflammation, and immunity, whereas in $X_{p}$, they focused on biomarkers associated with systemic immunity, inflammation, and metabolism [68]. The above-mentioned studies confirmed that AD is a very complex and multifactorial neurodegenerative disease.

Another proposed system for the interpretation of CSF biomarker results is the interpretive consensus of biochemical profiles of AD biomarkers based on data from 40 worldwide research centers [20]. Results from each clinical laboratory included control of pre-analytical factors [20]. This approach resulted in a standardized commentary for eight biomarker profiles [20]. Each profile included β-amyloid level (Aβ 1-42 or Aβ 1-42/Aβ 1-40 ratio), total tau (t-tau) and p-tau(181) scores which take a binary score of normal (N) and pathological (P) [20]. By way of illustration, profile PPP—amyloid (P), t-tau (P), p-tau(181) (P)—has been described as: “Biochemical profile consistent with Alzheimer’s disease” or PNN has been described as: “Biochemical profiles consistent with an amyloidopathy” [20]. The interpretive consensus will allow for comparison of patient outcomes in the future and may enable standardization of the reporting of results. Possible interpretations of biomarker results in different score systems were collected and are presented in Table 1.

Early detection and diagnosis of AD remains a challenge. However, AD biomarkers show high diagnostic accuracy and sensitivity at the MCI stage of the disease, which is highly nonhomogeneous and can have many causes [4,16]. Cognitive impairment is not typical of older age, but may result from head trauma, metabolic disorders or substance abuse. In patients who have already progressed to MCI due to AD, the most common clinical manifestations are short-term memory impairment, anomia, and speech and language difficulties [16,17]. All symptoms are caused by neuropathological changes that can be monitored by in vivo biomarkers [69]. Researchers primarily use neuropsychological tests and biochemical biomarkers, which may be applied in specialized clinical settings, to help determine possible causes of MCI symptoms. Some patients with MCI will progress to full-blown AD [8]. Therefore, monitoring of the combination of tTau, pTau181, Aβ 1-42 and Aβ 1-42/Aβ 1-40 has proved to be very important in estimating changes in biomarker concentrations at baseline and after 4–6 years of follow-up [70,71]. Interestingly, the highest baseline concentrations of classical biomarkers, such as CSF Tau and Aβ 1-42, in MCI patients have been shown to be strongly associated with subsequent progression to AD (hazard ratio (HR) 17.7, *p* < 0.0001) [72]. The same study revealed that the use of Tau and the Aβ 1-42/pTau181 ratio had very similar diagnostic utility (sensitivity 95%, specificity 87%, HR 19.8) [72]. The results of the study are consistent with other multicenter studies, which have demonstrated that core AD CSF biomarkers, particularly the combination of low CSF Aβ 1-42, and high CSF tau and ptau181, can accurately predict progression from MCI to AD dementia (i.e., prodromal AD) [73,74]. These findings have allowed for the application of core AD biomarkers in diagnosing MCI in research and clinical settings [16]. While studies on AD and MCI have appropriate and specific diagnostic categories, the preclinical stage is still debated [75].

## 5. Preclinical Stage of Alzheimer’s Disease

**Figure 2:**
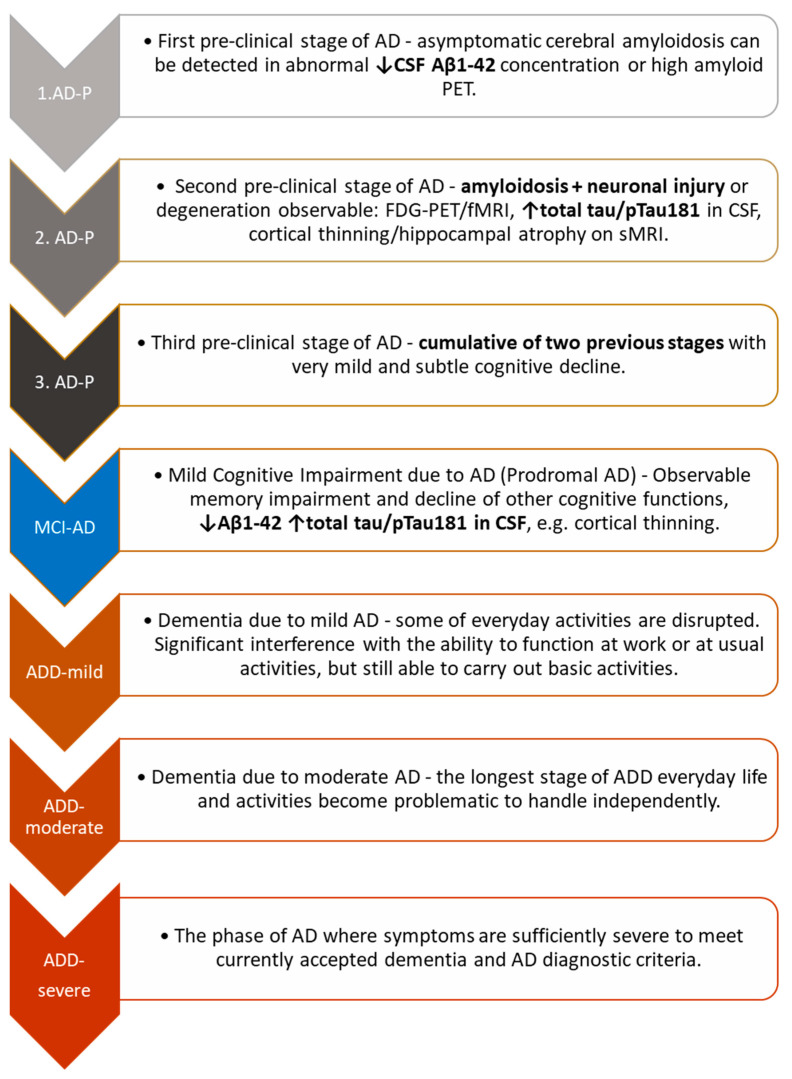
Alzheimer Disease continuum. Abbreviations: AD-P, preclinical stages of Alzheimer’s disease; AD, Alzheimer’s disease; MCI-AD, mild cognitive impairment due to Alzheimer’s disease; CSF, cerebrospinal fluid; FDG, fluorodeoxyglucose; PET, positron emission tomography.

The establishment of biomarkers have shifted diagnosing AD from dementia to the prodromal and nonsymptomatic stage [6,76]. CSF biomarkers allow for the detection of pathological changes before the onset of cognitive symptoms with high accuracy, sensitivity, specificity and have potential utility in preclinical diagnosis [16]. The preclinical stage of AD is still extensively debated by various consortia and workgroups [15,18]. The Diagnostic Guidelines for Alzheimer’s Disease proposed by the National Institute on Aging, NIH and Alzheimer’s Association, Chicago (https://www.alz.org, accessed on 6 June 2022) have been expanded to include three additional stages in the preclinical phase of the disease (Figure 2) [21]. Based on biomarker results, preclinical AD can be recognized. However, it can be used only for scientific research, not in clinical practice [6,15,18]. On the one hand, positive biomarker results in the early stages of the disease indicate that the pathological processes have already begun. On the other hand, these processes are not so advanced as to manifest themselves in everyday life, such as impairment of cognitive functions, nor is there certainty that progression will occur. The application of CSF or PET biomarkers in the diagnostic process allows for the detection of amyloidosis a number of years before manifestation of symptoms.

The risk of progression from the preclinical stage to MCI due to AD may depend on several factors, such as age, the female gender, presence of the apolipoprotein E4 (APOE4) variant and presence of CSF biomarkers. The preclinical AD stage may vary between individuals for several reasons, but age of onset is one of the most critical risk factors. It is probable that not every patient with preclinical AD pathology develops MCI or AD dementia. As Vermunt et al. noted, the estimation of disease duration becomes more accurate if age, sex, clinical status, APOE and abnormal Tau in CSF are included [77]. The conclusion seems to be supported by the study of Cho et al., which demonstrated that a significant pattern of progression from preclinical AD to MCI due to AD was 7.8 years and to AD dementia was 15.2 years [70]. The progression model was developed based on the Amyloid biomarker in PET scans and APOE4 in preclinical research and estimated Alzheimer’s Disease Assessment Scale-Cognitive Subscale 13 (ADAS-cog 13) scores [70]. In a different study, a more rapid rate of progression to MCI or AD was observed in individuals with preclinical AD (cognitively normal with positive AD biomarkers) in comparison to biomarker-negative individuals. Furthermore, progression rates differed between different preclinical stages of AD, where stage 3 developed more rapidly than stage 2, and stage 2 developed more rapidly than stage 1 [6]. These results further emphasize the rationale for conducting preclinical phase studies due to the potential application of therapy as early as the first stage of the disease. However, detectability of pathological changes in the preclinical stage based on CSF biomarkers is hindered by the absence of a reason to collect CSF from patients.

## 6. Legal Aspects of Lumbar Puncture

Some medical procedures, including the collection of cerebrospinal fluid by lumbar puncture, may involve a degree of risk for the patient, and are, therefore, subject to criminal law. In Polish criminal law, the granting of informed consent by patients to undergo diagnostic or therapeutic procedures is required, while performance of tests or administration of treatment without the patient’s consent is a crime [1]. However, under special conditions, such consent may also be provided by another person, e.g., the patient’s caregiver, who has the authority to make decisions for the patient. Such a situation may apply to dementia patients who are not able to make their own decisions at an advanced stage of the disease. The regulation of the patient’s informed consent for diagnostic procedures, such as a lumbar puncture, is a very important issue concerning the doctor–patient relationship as it defines the limits of the rights of the person performing therapeutic activities towards the patient and indicates the doctor’s basic duties in the treatment process. On the other hand, the right to make informed decisions about treatment protects the patient’s fundamental interests and clearly defines his or her rights. The patient’s participation in the treatment process consists in making conscious decisions about the treatment by a person without medical knowledge on the basis of information provided by the doctor. Moreover, this right is directly related to the doctor’s duty to inform the patient about his or her health condition. However, if in some situations it is not possible for the patient to provide his or her informed consent, such a decision is usually made by a court of law. In such cases, the judge appoints other people to make such decisions on behalf of the patient. Depending on the situation, these people may be parents, carers or legal guardians of the individual concerned.

## 7. Recommendations and Challenges

An early diagnosis allows the patient, their family members and doctors to develop care plans, select the most appropriate treatment and understand factors that increase the risk of progression [76]. The prevalence of AD increases with age, and ageing populations appear to be a global public health challenge [1]. Epidemiological data indicate that people who develop AD dementia are 65 or older. This type of dementia is known as late-onset Alzheimer’s disease (LOAD). Similarly to other common chronic diseases, AD develops as a result of an interplay between multiple factors. The APOE-e4 gene has the most significant impact on the risk of developing LOAD. The APOE-e4 plays an essential role in cholesterol transportation through the bloodstream, reduces the clearance of amyloid-beta plaques and performs a number of other functions. The second important risk factor for AD is age. The percentage of people with AD grows exponentially with age: 5.3% of those aged 65 to 74, 13.8% of those aged 75 to 84, and 34.6% of those aged 85 and older have AD [1]. Examples of modifiable risk factors include lifestyle and physical activity, smoking, education, comorbidity, blood pressure and diet. Recommendations from the Lancet Commission on dementia prevention, intervention and care in 2020 suggest that addressing modifiable risk factors could prevent or delay the onset of up to 40% of dementia cases [78]. Prevention and planning of therapeutic strategies are more promising when diagnosis is made early [76]. One of the possible solutions that can effectively reduce the risk of AD dementia could be very early therapeutic intervention. However, to make it possible, screening tests, complemented by CSF or PET biomarker results, would be needed. Advances in the development of ultrasensitive methods increasingly allow for the testing of these core biomarkers in blood (plasma or serum). Particularly promising results were obtained in studies investigating the concentrations of biomarkers, such as pTau181, pTau217 and pTau231, in AD patients [79,80,81]. Although the sensitivity and specificity of these biomarkers do not yet match those of CSF biomarkers, the results are dependent on the methods used [80]. The development of tests based on blood biomarkers is crucial for screening older adults. However, to measure these biomarkers, ultrasensitive methods are needed. It is also important to note that using CSF and neuroimaging biomarkers provides the earliest and most reliable clinical picture. The psychological tests and criteria (DSMIV, DSMV, ICD10 or ICD11) are based only on cognitive symptoms and can provide important information regarding performance of activities of daily living. In summary, the use of CSF biomarkers and neuroimaging tests allows for an accurate and early diagnosis, based on well-established diagnostic criteria, which improves patient outcomes.

## 8. Conclusions

It is considered that the most accurate diagnosis of AD dementia requires the application of neuropsychological tests, CSF and neuroimaging biomarkers [1,4,13]. Omission of any of the stages may impact diagnostic sensitivity and specificity. Some data indicate that neuroimaging and CSF biomarkers are closely correlated [13]. Many studies suggest that classical CSF biomarkers have the highest clinical value in the diagnosis of AD. Additionally, they also correlated with PET biomarkers and cognitive decline [4,13,67]. The general trend in diagnostic testing is toward the earliest possible detection of disease with the lowest risk of CSF collection, and a reduction in the cost of testing. It is also important to emphasize that the development of ultrasensitive techniques and research on new biomarkers by scientists from interdisciplinary centers may allow for improvement in early diagnosis as well as enable the search for novel therapeutic targets [82].

## Figures and Tables

**Table 1:** Comparison between different interpretation scales and scores for highly probable AD, improbable or not inconsistent and healthy individuals. Abbreviations: A+ positive amyloid concentration, A− negative amyloid concentration, T+ positive results of tau concentration, T− negative results of tau concentration, (N)+ positive neurodegeneration, (N)− negative neurodegeneration, first P—positive amyloid concentration, second P—positive total tau concentration, third P—positive pTau181 concentration.

Scales of AD Biomarkers			
Amyloid pTau181/tTau	Erlangen Score [63]	AT(N) [62]	Harmonized Report [20]
	Score = 2	A+	P
	Score = 3	T+	P/P
Possible results of AD patients	Score = 4	A+T+(N)+ or A+T-(N)+ or A+T+(N)−	PPP
AD improbable [2]/not inconsistent [4]	Borderline score of one type biomarker = 1	A-T+(N)− or A-T-(N)+	NPN or NNP
Results of healthy individuals	0—‘no neurochemical evidence for AD’	A-T-(N)− ‘Normal AD biomarkers’	NNN—‘Biochemical profile not consistent with Alzheimer’s disease’

## Data Availability

Not applicable.
